# New Appliances and Things Medical

**Published:** 1895-09-21

**Authors:** 


### NEW APPLIANCES AND THINGS MEDICAL.

### COGNAC: THREE STAR BRAND.

(T. and F. Martell.)

We have been forwarded a sample of this brandy; although the brand is known to ourselves as well as the rest of the world we have made renewed trial of it in certain cases in which stimulants were indicated. The therapeutic effect has been all that could be desired, and the patients have, one and all, expressed great appreciation of the fineness and flavour of the spirit. Its age is such as to ensure the elimination of all deleterious products of distillation.

